# Treatment cost and costing model of obstetric complications at a hospital in Myanmar

**DOI:** 10.1371/journal.pone.0213141

**Published:** 2019-03-20

**Authors:** A. Thi Win Shwe, Arthorn Riewpaiboon, Usa Chaikledkaew, Sitaporn Youngkong

**Affiliations:** 1 Division of Social and Administrative Pharmacy, Department of Pharmacy, Faculty of Pharmacy, Mahidol University, Bangkok, Thailand; 2 Department of Pharmaceutics, University of Pharmacy, Yangon, Ministry of Health and Sports, Myanmar; Case Western Reserve University, UNITED STATES

## Abstract

Maternal health still remains a major challenge in almost all developing countries. In Myanmar, the country met only 62% of its target for the maternal mortality rate (130 per 100,000 live birth) even though proportion of skilled birth attendant (SBA) and antenatal care (ANC) coverage was 80% in 2015. Despite the estimated large maternal complications, most maternal healthcare program ignored the burden of those morbidity because of limited understanding of the incidence and prevalence of morbidity conditions and cost of those morbidity burdens on society. The present study provides a general idea of the scope of obstetric complication, incidence of obstetric complication, and cost of those morbidity burdens on society. We conducted a retrospective incidence-based cost of illness study related to obstetric complication from the healthcare system perspective at 25 bedded township hospital in Yedashae during the fiscal year of 2015–2016. For the cost of obstetric complication, average treatment cost was 26.83 USD (±8.59). When looking by disease category, average treatment cost for incomplete abortion was 35.45 USD (±1.75); pelvic inflammatory disease (PID) was 16.01 USD; pregnancy-induced hypertension (PIH) was 21.02 USD (±4.68); ante-partum hemorrhage (APH) was 14.24(± 0.25); post-partum hemorrhage (PPH) was 27.04 USD (±1.56); prolonged labor was 37.55 USD (±0.42); and septicemia was 16.51 USD (±2.15). Significant predicting variables in obstetric complication cost model were incomplete abortion, prolonged labor, post-partum hemorrhage (PPH), pregnancy induced hypertension (PIH), patient age and septicemia. From this study, we can summarize the most frequently occurred obstetric complication in that township area, actual cost burden of those complications and obstetric complication cost model which can be useful for hospital financial management. This study can be considered as a starting point for cost of illness analysis in Myanmar to prioritize and target specific health problem at a country level for policy maker to set priorities for health care intervention.

## Introduction

Efficiency of health care system is important in developing countries that are struggling to improve public health with limited fund. This has become more vital in services related with maternal health which still remain major challenges in almost all developing countries [1].

Obstetric complications usually consider to health problems related to pregnancy and delivery and we can also use the term, “maternal morbidity.” Maternal morbidity is generally defined as any illness or injury caused by or associated with pregnancy or child birth [2]. It also stands as a group which constitute one of the leading causes of the burden of disease for women of reproductive age throughout the world and contribute to high level of mortality and disability in developing region [3].

In 2006 WHO report, more than half a million women died each year because of pregnancy and child birth; millions more become ill or disabled around the world. About 99% of these deaths occurred in developing countries of Africa and Asia. Also, one study in 4 developing countries showed that 58% - 80% of pregnant women developed acute health problem because of pregnancy and of which, 8% - 29% went on to develop chronic health problem [2].

Some reasons of high risk of pregnancy related complication in developing countries are: poverty, social and cultural prejudices, gender-based violence, lack of education and lack access to essential health care facilities. Also cost, distance and quality of service, cultural barriers and barriers related to knowledge and education become some obstacle to use essential MCH services [2].

In Myanmar, maternal health problems still stands as one of the major challenging problems. So, demand side financing maternal and child health voucher scheme (MCHVS) was implemented in Yedashae Township as a 3-year pilot program since 2013. Yedashae township is situated in Bago (east) province. Total population of Yedashae township was 209,217 (195,885 in rural area and 13,332 in urban area), total delivery patients were 4,222 and ANC coverage was 91% according to 2014 data. It has one 25-bedded township hospital, 1 MCH center, 3 station hospital, 1 station health unit and 7 rural health center (RHC). Although it is not very far from Nay Pyi Taw, capital city of Myanmar which was only 40 miles away, its socioeconomic status is low [4].

In MCHVS program, voucher distributers distributed vouchers to pregnant mother which included maternal and child health (MCH) benefit package comprising four ante-natal care (ANC) visits, normal delivery with SBA at home or at health center, and one post-natal care (PNC) visits. Pregnant mother with voucher can receive MCH services from health facilities for free and they also can get incentive for receiving those services. The main objectives of this programs were to reduce financial barrier for receiving MCH service at the primary level and to attract pregnant women from that area to receive MCH services by giving incentive [5].

Still, in 2015, the country met only 62% of its target for the maternal mortality rate (130 per 100,000 live birth) even though proportion of SBA was 80% and antenatal care (ANC) coverage was 80% in 2015 [6, 7]. To reduce maternal mortality, we need to evaluate current maternal and child health (MCH) services and implement program to improve MCH services.

Despite the estimated large maternal complications, most maternal healthcare program has done little to reduce the burden of those morbidity, which affect the large proportion of women. This gap is because of our limited understanding of the incidence and prevalence of morbidity conditions and cost of those morbidity burden on society [3].

The concept of cost of illness (COI) study is to estimate the economic burden of illness that imposes on society. Information from COI is helpful to formulate and prioritize healthcare policies and interventions and eventually allocate healthcare resources with budget constraints in order to achieve policy efficiency [8].

The objective of this cost of obstetric complication study was to support evaluation of that MCHVS program through understanding cost of obstetric complication burden and intended to get an idea of how much cost we can save if MCHVS program can reduce those complications. Therefore, this study can give empirical evidence of obstetric complication cost in that area to hospital administrator and also policy maker. Moreover, this study can provide general idea of the scope of obstetric complication, incidence of obstetric complication, and cost of those morbidity burden on society; even though this cannot be representative for whole country.

## Materials and methods

### Study design

We conducted a retrospective incidence-based cost of illness study related to obstetric complication from the healthcare system perspective. Incidence-based cost of illness is a technique used for measuring economic burden of patients from the onset to the end of illness [9].

### Study area

We selected 25-bedded township hospital in Yedashae Township as this study is part of economic evaluation of maternal and child health voucher scheme (MCHVS) program which is implemented as a pilot program in this study township [5].

### Ethical approval

Ethical clearance for the record review at study hospital was obtained from the ethical review committee of the Department of Public Health, Ministry of Health and Sports (MOHS), Myanmar as part of the larger study, social return on investment of maternal and child health voucher scheme (MCHVS) program in Yedashae Township, Myanmar. Ethical review committee waived the requirement of informed consent and all data were fully anonymized by using only patient registration number (coding).

### Study sample

As a study sample, patients with obstetric complication belong to all age groups who received treatment during 2015–16 fiscal year were included in this study. We included all obstetric complications that we found during data collection period (2015~2016 fiscal year) in study hospital. Firstly, we reviewed hospital inpatient registration book from inpatient department to select cases of obstetric complications. From the result of reviewing hospital inpatient registration book, we defined scope of obstetric complication into 7 categories. They are incomplete abortion, pelvic inflammatory disease (PID), pregnancy induced hypertension (PIH), antepartum hemorrhage (APH), post-partum hemorrhage (PPH), prolonged labor and septicemia. Disease diagnosis was based on the diagnosis described in specific patient record chart. Since the standard disease classification system such as ICD 10 was not experienced in most of the hospitals of Myanmar especially small hospital like Yedashae township hospital, medical doctors from those hospitals just made diagnosis based on their personal experience.

### Data collection

One year data for all obstetric complications occurred in that study hospital which was from April 2015 to March 2016 (2015 fiscal year), were collected retrospectively from the study hospital. We selected cases from patient registration book of inpatient department for the whole study year based on our scope of obstetric complication and then we reviewed case by case of our selected inpatient medical record chart to get the patients’ demographic characteristics, type of obstetric complication, types and quantities of medical services received, types and quantities of medical supplies used. In demographic data, we classified as rural and urban according to their geographic location. If patient was from Yedashae town itself, we classified as from urban area and if from nearby village under Yedashae township, classified as rural area.

### Costing method

Generally, cost of illness study is conducted by summing up of direct medical cost, direct non-medical cost and indirect cost but it also depends on perspective of the study [9]. For patient perspective, it includes direct medical cost (charges they need to pay for drugs and medical services related to their illness), direct non-medical cost (travel, meal and hotel charges for both patient and caregivers, charges for equipment and facilities for patient and time loss of caregiver in terms of income loss) and indirect cost (time loss of patient due to treatment and recovery, time loss of patient due to death or permanent severe disability in term of income loss). For healthcare system/ hospital perspective, it only considers on direct medical cost such as drugs and medical services provided from hospital. From society perspective, it considered as a whole of direct medical cost from healthcare system/ hospital perspective and direct-non medical cost together with indirect cost from patient perspective. [10].

As our study is from healthcare system perspective, we focused our analysis on direct medical cost employing the bottom up or micro-costing approach that estimate treatment cost (direct medical cost) by summing all drugs used (drug cost) and medical services (laboratory cost, routine service cost and operation cost)received by an individual patient [2, 10]. Drug cost included drugs used in obstetric complication from individual patient chart such as antibiotic, normal saline, spirit, cotton, glove, etc; laboratory cost included grouping and matching, complete picture (CP), HIV test, HbsAg test, etc; routine service cost included OPD cost, IPD cost, etc; and operation cost for caesarean delivery. For individual patient cost calculation, we used type of complication, services utilization such as number of hospitalization days, laboratory tests used, X-ray used, types and numbers of medical services received and drug utilization data such as types and number of drugs used from the individual patient case record chart which was collected from inpatient department.

To calculate drug cost, we use invoice which included quantity and purchasing price of individual drugs from pharmacy department. To calculate cost of each medical service, we multiplied quantities of medical services used for each patient and their unit cost. Cost related to time of health professionals and hospital recurrent expenses are already included in unit cost of medical services as labor cost and material cost because we considered economic cost in unit cost calculation which already included capital, labor and material cost for each service. So, to avoid double counting, we didn’t consider separately for those costs in this study. Then, we summed up the cost of all medical services (laboratory cost, routine services and operation cost) and drugs (drug cost) of each patients to obtain individual treatment cost (direct medical cost). After that, we summed up for the whole patient to get total treatment cost (total direct medical cost) of all patients in terms of general obstetric complication and complication by category.

For unit cost, we calculated unit cost of medical services given by study hospital in economic approach by using standard costing method. According to function of department, cost centers were divided into 5 absorbing cost centers (ACCs) and 2 transient cost centers (TCCs). Direct cost of each cost center was calculated by summing capital cost which included annualized economic cost with 3% discount rate, labor cost and material cost. Indirect cost which was equal to the cost of TCCs, was allocated to ACCs via simultaneous equation method. Outputs of TCCs were chosen as allocation criteria. The direct and indirect cost of each ACCs were then added to get full cost of each ACCs. To calculate unit cost, we used average cost method and micro costing method based on availability of data [10].

### Analysis of data

Data extraction, management and costing analysis are done by using the computer software Microsoft Excel 2016. All costs were obtained and presented in the monetary unit (USD) in 2015 fiscal year which was converted from local currency, Myanmar Kyat (MMK) by using average exchange rate of 2015 fiscal year from maximum and minimum exchange rate throughout 2015 fiscal year(1 USD = 1199.07MMK) [11]. For statistical analysis, we used SPSS version 21 software. Demographic information was presented by frequency and proportion. We calculated means with standard deviation (SDs) for continuous variables.

We also used multiple linear regression (MLR) analysis applying step-wise method [12] to find the significant predicting factors (independent or continuous variable) influencing total treatment cost (dependent variable) and to construct the forecasting model. Independent variables considered in this cost function model included age (year), distance from hospital (geographic location: rural and urban), receiving ANC (yes, no) and type of complication. All types of complication were recorded as dummy variable except reference case. For reference case, we chose from existing type of complication based on least treatment cost. In our study, ante partum hemorrhage (APH) is least treatment cost compared with other complication and therefore we chose it as a reference case. We excluded length of stay as a predictable variable because it cannot give strong evidence for cost saving. Impact of length of stay on quality of service is unclear to use it as an efficiency indicator. Despite increased efficiency, short length of stay can be adverse effect on treatment in term of increasing readmission rate, transferring cost to other part of health sector or onto patient and their family [13]. A step-wise method was applied for that analysis. A model was constructed to estimate treatment cost of each complication. In model, independent variable with a probability value of F statistics ≤0.05 were entered for analysis. Model assumption and diagnostic test were examined by testing linear relationship, normal distribution, homoscedasticity, multicollinearity, influential observations, and outliers [12, 14].

## Results

### Demographic data of hospitalized patients

From one year data (2015 fiscal year) of Yedashae township hospital, there was a total of 91 cases of obstetric complication, which consists of 26 cases for incomplete abortion (28.6%); only one case for pelvic inflammatory disease (PID) (1.12%); 23 cases for pregnancy induced hypertension (PIH) (25.3%); 11 cases for ante -partum hemorrhage (APH) (12.1%); 17 cases for post-partum hemorrhage (PPH) (18.7%), 9 cases for prolonged labor (9.9%) and 4 cases for septicemia (4.4%). Overall total mean age was 26.75(±5.09) ranging from 19 to 39 years. We also explored according to age group for frequency of overall complication and we found that 64 complication cases (70.3%) were from<30 year group and 27 cases (29.7%) from ≥ 30 year group.

When we looked at their distance from hospital, we classified as urban area and rural area which showed (31 cases, 34.1%) and (60 cases, 65.9%) respectively. And in case of receiving ANC service, (14 patients, 15.4%) didn’t receive ANC services during their pregnancy period and (77 patients, 84.6%) received ANC. Average length of hospital stay for overall complication is 3.12 days and median is 3 days ranging from 2 days to 5 days.

From those results, we can see that obstetric complication was highest in <30 age group from rural area in Yedashae township. Highest risk of obstetric complication by category was incomplete abortion which stood 28.6% among 7 obstetric complication followed by pregnancy induced hypertension (PIH) which was 25.3%. We summarized demographic data for those results in Table 1.

**Table 1:** Demographic data of hospitalized patients with obstetric complication in Yedashae township hospital (2015 fiscal year).

Variables	Patients with obstetric complication (n = 91)
**Year (Age)**	
*Mean (±SD)*	26.75 (±5.09)
*Range (Min-Max)*	19–39
*<30*	64 (70.3%)
*≥30*	27 (29.7%)
**Location**	
*Rural*	60 (65.9%)
*Urban*	31 (34.1%)
**Length of stay (day)**	
*Mean*	3.12
*Medium*	3
*Range*	2–5
**Receiving ANC during pregnancy**	
*Yes*	77 (84.6%)
*No*	14 (15.4%)
**Type of complication**	
*Incomplete Abortion*	26 (28.5%)
*Pelvic Inflammatory Disease (PID)*	1 (1.1%)
*Pregnancy included Hypertension (PIH)*	23 (25.3%)
*Ante-Partum Hemorrhage (APH)*	11 (12.1%)
*Post-Partum Hemorrhage (PPH)*	17(18.7%)
*Prolonged Labor*	9 (9.9%)
*Septicemia*	4 (4.4%)

### Estimation of cost

#### Unit cost

In 2015, total hospital cost was 88,457 USD. When we broke down the cost as capital, labor and material cost, capital cost occupied 25.59%, labor cost occupied 51.25% and material cost occupied 23.16%. We calculated 26 basic medical service cost which is shown in Table 2.

**Table 2:** Unit cost of medicals (USD in 2015 values).

Name of basic medical service	Unit cost
Operation	10.37
Out Patient Department (OPD) visit	1.60
Inpatient Department (IPD); admission	9.48
Inpatient Department (IPD); patient day	2.02
Adult Chest X-ray	6.97
Arms, Legs and Knee X-ray	6.74
Child X-ray	6.67
Human Immunodeficiency Virus (HIV) test	3.15
Hepatitis B Surface Antigen (HBs-Ag) test	0.56
Hepatitis C Virus (HCV) test	1.04
Venereal Disease Research Laboratory (VDRL) test	0.77
Hemoglobin (Hb) test	0.25
Complete Picture (CP) test for blood	1.67
Randon Blood Sugar (RBS) test	0.40
Erythrocyte Sedimentation Rate (ESR)	4.07
Packed Cell Volume (PCV) test	0.43
Bleeding Time (BT), Clotting Time (CT)	0.75
Grouping and Matching (G & M)	0.42
Rh Blood Group Test	0.40
Malaria Parasite (MP) test (with Film)	1.22
Malaria Parasite (MP) (With Immunochromatographic Assay (ICT))	1.03
Ziehl-neelsen stain (Z-N stain) test	1.06
Urine Re	1.38
Urine Re (test strip)	0.44
Urine Chorionic gonadotrophin (UCG) test	0.11
Stool Re	0.66

1 USD = 1,199.07 MMK in 2015 average exchange rate, NA = Not Applicable

#### Total treatment cost (total direct medical cost)

We calculated total treatment cost of obstetric complication in term of general obstetric complication and complication by category by age group during our study period. During the study period, we found 91 cases of all obstetric complication for all age group. Total treatment cost of all obstetric complication was 2,441.56 USD (age group <30 used 1,711.99 USD and ≥ 30 used 729.57). When we break down by disease category, incomplete abortion was 921.74 USD (age group <30 used 736.76 USD and ≥ 30 used 184.98 USD); pelvic inflammatory disease was 16.01 USD (only one case for the whole year from <30 group); pregnancy induced hypertension (PIH) was 483.46 USD (age group <30 used 217.32 USD and ≥ 30 used 266.14 USD); ante-partum hemorrhage (APH) was 156.59 USD (age group <30 used 98.86 USD and ≥ 30 used 57.73 USD); post-partum hemorrhage (PPH) was 459.73 USD (age group <30 used 407.32 USD and ≥ 30 used 52.41 USD); prolonged labor was 337.99 USD (age group <30 used 187.90 USD and ≥ 30 used 150.09 USD); and septicemia was 66.04 USD (age group <30 used 47.81 USD and ≥ 30 used 18.23 USD).

**Fig 1:**
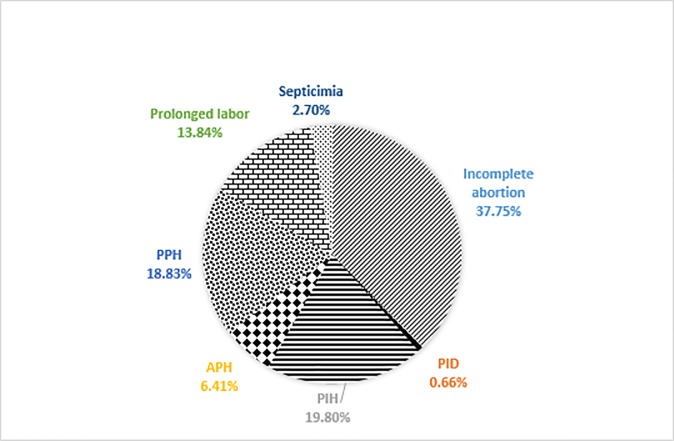
Percentage of total treatment cost by disease category (2015 fiscal year).

From those results, we can see that total treatment cost of incomplete abortion was the largest portion which stood 37.75% followed by pregnancy-induced hypertension (PIH) which stood 19.80%, then the third one was post-partum hemorrhage (PPH)which was 18.83%, the fourth one, prolonged labor, was 13.84%, the fifth one, ante-partum hemorrhage (APH), was 6.41%, the sixth one, septicemia, was 3.42% and final one was pelvic inflammatory disease (PID) which was 0.66%. To be understandable, we also presented percentage of total treatment cost by disease category with Fig 1.

#### Average treatment cost (direct medical cost)

We also calculated average treatment cost of obstetric complication by age group in terms of general obstetric complication and complication by category. Individual average treatment cost of obstetric complication for all age was 26.83 USD (± 8.59) whereas for <30 years age group, cost was 26.75 USD (± 8.76) and ≥ 30 age group, cost was 27.02 USD (±8.34).

When we looked at by disease category, average treatment cost of incomplete abortion was 35.45 USD (±1.75) [35.08 USD(±1.70) for <30 group and 37 USD(±0.98) for ≥ 30 age group]; pelvic inflammatory disease (PID) was 16.01 USD which occurred only one case during our study period which is from <30 age group; pregnancy-induced hypertension (PIH) was 21.02 USD(±4.68) [18.11 USD(±4.33) for <30 group and 24.19 USD(±2.50) for ≥ 30 age group]; ante-partum hemorrhage (APH) was 14.24(± 0.25) [14.12 USD(± 0.24) for <30 group and 14.43 USD(± 0.12) for ≥ 30 age group]; post-partum hemorrhage (PPH) was 27.04 USD (±1.56) [27.15 USD(±1.63) for <30 group and 26.21 USD(±0) for ≥ 30 age group]; prolonged labor was 37.55 USD(±0.42) [37.58(±0.43) for <30 group and 37.52(±0.47) for ≥ 30 age group]; and septicemia was 16.51 USD(±2.15) [15.94 USD(±2.23) for <30 group and 18.23 USD (±0) for ≥ 30 age group].

From those results, we can see that average treatment cost was highest for prolonged labor and then decreased with the order of incomplete abortion, PIH, PPH, septicemia, PID and APH. by disease category.

Table 3 presents total and average treatment cost for general obstetric complication and complication by disease category.

**Table 3:** Total and average direct medical cost by disease category (USD) (Mean ± SD, medium).

Complication by category	Average Treatment Cost/Case															Total treatment Cost (Total Direct Medical Cost)
	Drug cost			Laboratory cost			Routine service cost			Operation theatre cost			Average Treatment Cost (Average Direct medical cost)			
	Mean	SD	Median	Mean	SD	Median	Mean	SD	Median	Mean	SD	Median	Mean	SD	Median	
***Incomplete abortion (n = 26)***	10.42	1.04	10.36	9.38	0	9.38	5.27	1.00	6.05	10.37	0	10.37	35.45	1.75	35.45	921.74
Age <30 (n = 21)	10.24	0.99	10.30	9.38	0	9.38	5.09	1.03	6.05	10.37	0	10.37	35.08	1.70	34.87	736.76
Age≥30 (n = 5)	11.19	0.98	10.78	9.38	0	9.38	6.05	0	6.05	10.37	0	10.37	37	0.98	36.59	184.98
***PID (n = 1)***	7.96	0	7.96	0.40	0	0.40	7.65	0	7.65	0	0	0	16.01	0	16.01	16.01
Age <30 (n = 1)	7.96	0	7.96	0.40	0	0.40	7.65	0	7.65	0	0	0	16.01	0	16.01	16.01
Age≥30 (n = 0)	0	0	0	0	0	0	0	0	0	0	0	0	0	0	0	0
***PIH (n = 23)***	8.99	2.14	9.30	3.50	0.81	3.86	8.53	2.18	9.67	0	0	0	21.02	4.68	22.82	483.46
Age <30 (n = 12)	7.79	2.24	8.71	3.34	0.94	3.86	6.98	1.57	6.65	0	0	0	18.11	4.33	19.22	217.32
Age≥30 (n = 11)	10.30	0.96	10.07	3.67	0.63	3.86	10.22	1.30	9.67	0	0	0	24.19	2.50	23.61	266.14
***APH (n = 11)***	2.85	0.25	2.87	9.78	0	9.78	1.60	0	1.60	0	0	0	14.24	0.25	14.26	156.59
Age <30 (n = 7)	2.74	0.24	2.87	9.78	0	9.78	1.60	0	1.60	0	0	0	14.12	0.24	14.26	98.86
Age≥30 (n = 4)	3.05	0.12	3.10	9.78	0	9.78	1.60	0	1.60	0	0	0	14.43	0.12	14.49	57.73
***PPH (n = 17)***	5.28	1.56	4.45	9.78	0	9.78	1.60	0	1.60	10.37	0	10.37	27.04	1.56	26.21	459.73
Age <30 (n = 15)	5.39	1.63	4.45	9.78	0	9.78	1.60	0	1.60	10.37	0	10.37	27.15	1.63	26.21	407.32
Age≥30 (n = 2)	4.45	0	4.45	9.78	0	9.78	1.60	0	1.60	10.37	0	10.37	26.21	0	26.21	52.41
***Prolonged labor (n = 9)***	11.35	0.42	11.67	9.78	0	9.78	6.05	0	6.05	10.37	0	10.37	37.55	0.42	37.87	337.99
Age <30 (n = 5)	11.38	0.43	11.67	9.78	0	9.78	6.05	0	6.05	10.37	0	10.37	37.58	0.43	37.87	187.90
Age≥30 (n = 4)	11.32	0.47	11.33	9.78	0	9.78	6.05	0	6.05	10.37	0	10.37	37.52	0.47	37.54	150.09
***Septicemia(n = 4)***	6.34	0.24	6.34	0	0	0	10.17	1.93	10.68	0	0	0	16.51	2.15	17.01	66.04
Age <30 (n = 3)	6.27	0.24	6.13	0	0	0	9.67	2.02	9.67	0	0	0	15.94	2.23	15.80	47.81
Age≥30 (n = 1)	6.54	0	6.54	0	0	0	11.69	0	11.69	0	0	0	18.23	0	18.23	18.23
***Total DMC (n = 91)***	8.07	3.11	9.16	7.55	3.24	9.38	5.29	3.11	6.05	5.93	5.16	10.37	26.83	8.59	26.21	2441.56
Age <30 (n = 64)	7.69	3.02	8.71	7.84	3.20	9.38	4.58	2.59	4.83	6.65	5.02	10.37	26.75	8.76	26.21	1711.99
Age≥30 (n = 27)	8.97	3.21	10.14	6.86	3.28	9.38	6.97	3.59	6.05	4.23	5.19	0	27.02	8.34	26.21	729.57

1USD = 1199.07 MMK in 2015 average exchange rate, PID = Pelvic Inflammatory Disease; PIH = Pregnancy Induced Hypertension; APH = Ante-partum hemorrhage; PPH = Post-partum hemorrhage.

#### Predicting factor for obstetric complication cost (obstetric complication cost model)

For obstetric complication cost model, all potential predictor variables used in this study are described in Table 4.

**Table 4:** Description of potential predictor variables (n = 91).

Variables
Age: mean = 26.75 year Distance from hospital: Rural = 65.9% Urban = 34.1% ANC: 84.6% Obstetric complications were recorded as dummy variables (Reference = Ante-partum hemorrhage (APH): 12.1%) Incomplete abortion (IA): 26 (28.6%) Pelvic inflammatory disease (PID): 1.1% Pregnancy induced hypertension (PIH): 25.3% Post-partum hemorrhage (PPH): 18.7% Prolonged labor (PL): 9.9% Septicemia: 4.4%

As the dependent variable, total treatment cost, was not normally distributed, we made transformation to natural logarithmic form. Then we analyzed with the original data (n = 91) to get the fitted model with the adjusted R^2^ = 0.854 (R^2^ = 0.863) and the probability of F test = 0.030. For assumption and diagnosis of model, it showed homoscedasticity, no multicollinearity and no influential observation [12]. But Durbin-Watson value which is used to test the independence of the residual was 1.154 which didn’t meet the criteria (criteria: 1.5 to 2.5) [15]. Also, the range of studentized deleted residual is from -4.698 to 2.165 (mean: -0.014) (criteria: < ±2) (12). It shows that there are some outliers in total treatment cost.

So, we cut extreme cases and analyzed again without extreme outlier by selecting the case with criteria range from -2 to +2 of studentized deleted residual. Fitted model without outlier (n = 87) is shown in Table 5 with the adjusted R^2^ = 0.954 (R^2^ = 0.958) and the probability of F test = 0.002.

**Table 5:** Fitted direct medical cost model (n = 87).

	Unstandardized Coefficients		T	Sig.	95.0% Confidence Interval for B	
	B	Std. Error			Lower Bound	Upper Bound
(Constant)	9.602	.048	198.724	.000	9.506	9.698
Incomplete abortion (IA)	.901	.025	35.808	.000	.851	.951
Prolonged labor (PL)	.947	.032	29.587	.000	.883	1.011
Post-partum hemorrhage (PPH)	.649	.028	23.450	.000	.594	.704
Pregnancy induced hypertension (PHI)	.407	.027	15.008	.000	.353	.460
Patient age	.006	.002	3.495	.001	.003	.009
Septicemia	.133	.042	3.206	.002	.051	.216

The significant predicting variables were Incomplete abortion, prolonged labor, post-partum hemorrhage (PPH), pregnancy induced hypertension (PIH), patient age and septicemia. For assumption and diagnosis of model, a scatter plot of residual against predicted value and all independent variable showed no funnel shape indicating homoscedasticity [12]. To test the independence of the residual, the model met the assumption that its Durbin-Watson value was 1.983 (criteria: 1.5 to 2.5) [15]. The condition index was the range of 1.000 to 14.211 (criteria: ≤ 30), tolerance was the range of 0.450 to 0.826 (criteria: >0.1) and variance inflation factor (VIF) was the range of 1.210 to 2.221(criteria <10). They indicated that there was no multicollinearity. Cook’s distance had a range of 0.000 to 0.293 (criteria:<1) which indicate no influential observation [12]. The fitted model equation is presented as follow:

LnTDMC=9.602+0.901IA+0.947PL+0.649PPH+0.407PIH–0.006Patientage+0.133Septicemia

We can estimate the expected response on the untransformed scale after fitting a linear regression model of a transformed scale by adjusting it with smearing factor. The smearing factor is the mean of anti-log (exponential form) of the unstandardized residuals [16]. The smearing factor of the fitted model (n = 87) was 1.0024.

By using smearing factor, we tested our fitted model by finding predicting cost of each significant obstetric complication from our model. We calculated for each complication by multiplying predictor variable and presence of complication. For patient age, we used mean age which is 26.75. Then, summation of each predicting costs was converted to anti-log form. After that, those costs were adjusted with smearing factor to get the final predicting cost for each significant complication. For the fitted value of each significant obstetric complication, we present in Table 6.

**Table 6:** Predicted cost of significant obstetric complication from fitted model.

Significant obstetric complication	Predicted cost (USD)
Incomplete abortion	35.75
Prolonged labor	37.43
Post-partum hemorrhage	27.79
Pregnancy induced hypertension	21.82
Septicemia	16.59

1USD = 1199.07 MMK in 2015 average exchange rate

## Discussion

### Cost of obstetric complication

From this study, we can estimate the most frequently occurred obstetric complication in that township area based on actual one year data from our study hospital. We summarized seven categories from our findings and among them incomplete abortion was the highest case which occupied 28.6% of all obstetric complication. Scope of obstetric complication in our study is in line with other countries as well as per WHO data. In 2006, WHO described 5 major obstetric complication which include hemorrhage, eclampsia, unsafe abortion, sepsis and obstructive labor [2].

When we looked at among the patients with obstetric complication, 65.9% of patients were from rural area. This can be because of the proportion of rural to urban ratio which is 14.7:1 at that township[4]. Moreover, in rural setting area, household and farm work is the vital work for women and they also cannot give proper care for their pregnancy because of not enough education of how to take care in terms of living style, food and medical care. All those reasons become the leading cause for incomplete abortion. Therefore, we should give health education up to grass-root level and also need to inform that they can get free MCH services at health care facilities.

Relatively, incomplete abortion occupied largest portion (37.75%) in total direct medical cost. When we see average treatment cost (average direct medical cost), overall general obstetric complication cost for all age was 26.83 USD (± 8.59). When we see by disease category, we can see that average treatment cost (average direct medical cost) was highest for prolonged labor, then decreased by incomplete abortion, PPH, PIH, septicemia, PID and APH in orderly. Cost of incomplete abortion in our study was 35.45 USD (±1.75). There is no study for cost of obstetric complications in Myanmar and even in other countries, only few studies which emphasized on incomplete/unsafe abortion can be found. One study in 2011 from Pakistan described that cost of incomplete abortion was ranged from 28 to 70 USD and other studies in 2009 from Africa and Latin America described cost of complication of incomplete abortion which was 83 USD and 94 USD respectively [17, 18]. Cost comparison across studies especially in cross countries are difficult and problematic because of difference in costing methods and resources availability [19].

When we break down average direct medical cost for general obstetric complication to drug cost, laboratory cost, routine service cost and operation cost, we can see that drug cost stood largest portion (33.19%); cost of routine service was the second largest (25.79%); laboratory cost was the third (25.37%) and operation theatre cost was the last (15.64%). For the second largest portion, routine service cost (OPD visit and IPD hotel stay), it also depends on study hospital’s efficiency. As our hospital bed occupancy rate (one of the hospital efficiency indicators) is only 41. 23% (WHO recommendation is 80%), it can be the reason for expensive routine service at our study hospital [20].

From our findings, we need to be concerned about incomplete abortion as it stood the most occurrence cause among all obstetric complication in that area and the highest percentage of cost occupied which is 37.75%. Also, when we looked in terms of its individual average treatment cost, it also stood second highest position.

### Obstetric complication cost model

Obstetric complication cost model can show significant predictor variable from healthcare system perspective. We analyzed with and without unacceptable outlier of cost (extreme cases) and compared. The R^2^ value is higher in second model without unacceptable outlier which show that it can predict more precise percentage. Also in part of model assumption, Durbin-Watson value of second model (for independence of residuals) met criteria whereas first model didn’t meet. So, we selected second model to predicted fitted value for significant obstetric complication. Even we can predict the cost of significant obstetric complication at that area from our estimating model, exact model equation cannot use at other hospital because of variation among hospital such as price variation of resources used, efficiency variation of resources used and output variance [16]. Despite those variations, other limitations such as small sample size, no standard treatment guideline for individual disease and no standardized disease classification system can vary model equation from one hospital to another.

If we take generalization as a concern, characteristics of hospitals need to be standardized in terms of price, treatment regimen and also diagnostic code basically. Anyway, cost model can apply hospital financial management tool by predicting obstetric complication cost to provide more accurate budget [21].

### Limitation

This study is based on 25-bedded township hospital under one township. Small sample size is the major limitation and since health facilities are not evenly distributed in Myanmar and utilization of healthcare services depend on the attitude of providers and quality of care, this study cannot be representative for the whole country. Second point is that the hospital didn’t used international standardized disease classification system and diagnosis is depend on only physicians’ experience. So, idea about standardizing treatment and controlling drug usage might be weak. Another point is reliance on estimated cost we used. Even we used unit cost data from the same hospital, treatment cost highly depends on the quality of those data. Since record keeping and health information system was poor, it can be over or under estimation of result for assumption data. Moreover, this is the first study for estimating cost of obstetric complication in Myanmar, we cannot compare pre-existing data to our study result. Despite these limitations, it can give the idea about cost of illness and the most frequently occurring category of obstetric complication at least at that township and we can use this study as a starting point for future cost of illness analysis.

## Conclusion

The present study provides the actual information about cost of obstetric complication and can summarize frequently occurring complication at that area. Even though there were some limitation, cost estimation of this study was done from actual treatment given in that hospital on an annual base rather than using theoretical estimation. So, we can estimate actual cost burden of illness on society at that township and use those data for hospital financial management even though it cannot be representative for the whole country. We can consider this study as a starting point for future cost of illness analysis in Myanmar and can go further comprehensive study from society perspective to prioritize and target specific health problem as a country level for policy maker to set priorities for health care intervention.

## Supporting information

S1 Table Patient record data & cost calculation file.xlsx. (XLSX)
